# MM2S: personalized diagnosis of medulloblastoma patients and model systems

**DOI:** 10.1186/s13029-016-0053-y

**Published:** 2016-04-11

**Authors:** Deena M.A. Gendoo, Benjamin Haibe-Kains

**Affiliations:** Bioinformatics and Computational Genomics Laboratory, Princess Margaret Cancer Center, University Health Network, Toronto, Ontario Canada; Department of Medical Biophysics, University of Toronto, Toronto, Ontario Canada; Department of Computer Science, University of Toronto, Toronto, Ontario Canada

**Keywords:** Subtype classification, Medulloblastoma, Diagnosis, Single-sample, Cancer, Mouse models, Primary tumours

## Abstract

**Background:**

Medulloblastoma (MB) is a highly malignant and heterogeneous brain tumour that is the most common cause of cancer-related deaths in children. Increasing availability of genomic data over the last decade had resulted in improvement of human subtype classification methods, and the parallel development of MB mouse models towards identification of subtype-specific disease origins and signaling pathways. Despite these advances, MB classification schemes remained inadequate for personalized prediction of MB subtypes for individual patient samples and across model systems. To address this issue, we developed the **Medullo-Model to Subtypes (*MM2S*)** classifier, a new method enabling classification of individual gene expression profiles from MB samples (patient samples, mouse models, and cell lines) against well-established molecular subtypes [Genomics 106:96-106, 2015]. We demonstrated the accuracy and flexibility of MM2S in the largest meta-analysis of human patients and mouse models to date. Here, we present a new functional package that provides an easy-to-use and fully documented implementation of the MM2S method, with additional functionalities that allow users to obtain graphical and tabular summaries of MB subtype predictions for single samples and across sample replicates. The flexibility of the MM2S package promotes incorporation of MB predictions into large Medulloblastoma-driven analysis pipelines, making this tool suitable for use by researchers.

**Results:**

The MM2S package is applied in two case studies involving human primary patient samples, as well as sample replicates of the GTML mouse model. We highlight functions that are of use for species-specific MB classification, across individual samples and sample replicates. We emphasize on the range of functions that can be used to derive both singular and meta-centric views of MB predictions, across samples and across MB subtypes.

**Conclusions:**

Our MM2S package can be used to generate predictions without having to rely on an external web server or additional sources. Our open-source package facilitates and extends the MM2S algorithm in diverse computational and bioinformatics contexts. The package is available on CRAN, at the following URL: https://cran.r-project.org/web/packages/MM2S/, as well as on Github at the following URLs: https://github.com/DGendoo and https://github.com/bhklab.

**Electronic supplementary material:**

The online version of this article (doi:10.1186/s13029-016-0053-y) contains supplementary material, which is available to authorized users.

## Background

Molecular subtyping is instrumental towards selection of model systems for fundamental research in tumour pathogenesis, and for clinical assessment of patients. To date, four molecular subtypes of Medulloblastoma (MB) have been established: SHH, WNT, Group3, Group4. The Group3 and Group4 MB subtypes are the least characterized, most aggressive, and have the poorest prognosis [1]. Model systems, including MB cell lines and genetically engineered mouse models [2], are being continually developed with the goal of studying MB subtype disease origins and signaling pathways. However, understanding the degree to which these model systems recapitulate Human MB subtypes remains the greatest challenge, especially for poorly characterized subtypes. In particular, many of the developed models have been predicted belong to the SHH subtype, with few models identified as recapitulating the Group3 or WNT phenotypes [3].

The lack of a versatile and personalized classification system hinders effective assessment of MB patients, and fundamental research into subtype-specific pathogenesis using model systems. To address these issues we developed a novel **Medullo-Model To Subtypes (MM2S)** classifier that matches individual gene expression profiles from MB samples against well-established molecular subtypes [4]. The MM2S algorithm is advantageous over existing MB-subtyping algorithms [3] by providing *single-sample* classifications while eradicating the need for a reference sample (e.g., human cerebellum) or sample replicates to generate predictions. MM2S design relies on a flexible, systems-based approach that makes it extensible and easily applicable across MB patients, human cell lines, and mouse models. We previously demonstrated *MM2S* extensibility and effectiveness across the largest meta-analysis of human MB patients, cell lines, and mouse samples to date [4]. In order to provide the scientific community with an easy-to-use and fully documented implementation of our flexible MB classifier we developed a new R package, **MM2S**, which implements the MM2S algorithm across human MB patients and model systems.

## Implementation

Training and development of the MM2S classification algorithm and hyperparameters has been previously described in detail [4], and the overall analysis design is provided in Additional file 1: Figure S1. Briefly, MM2S is trained on a set of 347 normal and tumor human MB samples pertaining to the SHH, Group3, and Group4 MB subtypes. Single-sample Gene Set Enrichment Analysis (ssGSEA) is conducted on mouse and human expression profiles using species-specific GMT files that were generated from common Gene Ontology Biological Processes (GO BP) genesets between human and mouse. Following ssGSEA, an ssGSEA-ranked matrix is generated from subtype-discriminative genesets by ranking genesets in descending order of their ES scores for each sample. To account for platform differences across test samples, we introduced an additional step that filters for common genesets between the test sample and human, prior to generating ssGSEA-ranked matrices for predictions. A k-nearest neighbor (KNN) classification uses the ssGSEA-ranked matrix and the 5 nearest neighbors of a given sample to make subtype predictions.

**Fig. 1:**
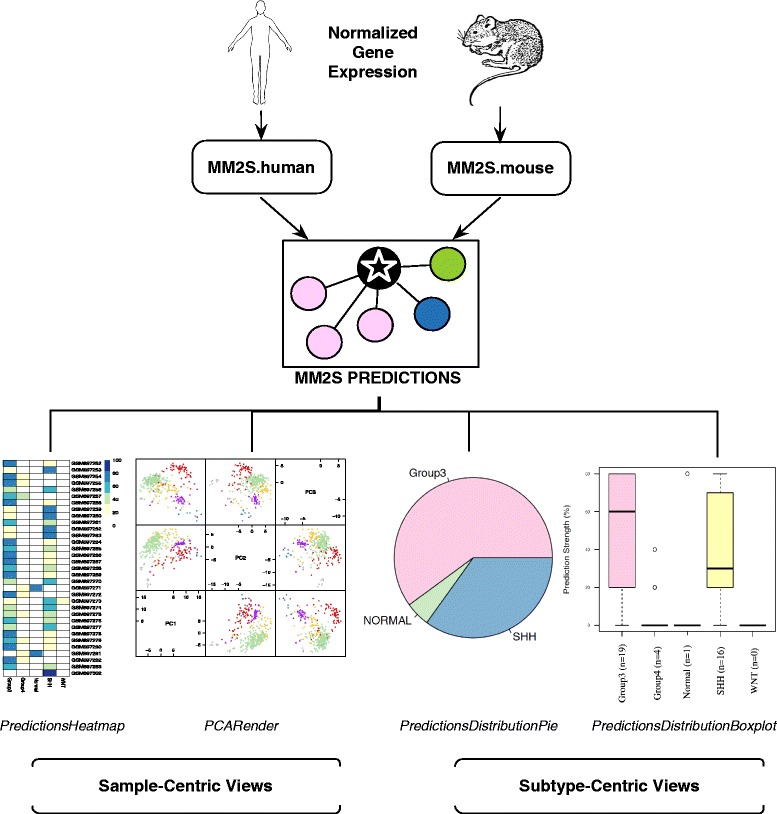
Overview of the MM2S package and its applications for MB subtypes of patient tumour samples and MB mouse models. A test sample (circled black star) representing normalized gene expression from human or mouse datasets is run using either of the *MM2S.human* or *MM2S.mouse* prediction functions, respectively. The MM2S prediction algorithm uses an ssGSEA and KNN-based approach to determine the MB subtype of a given sample, by looking at its 5 closest MB neighbors in 3-dimensional space. A selected number of functions can render the MM2S output in terms of sample-centric or subtype-centric views. The *PredictionsHeatmap* provides a heatmap representation of MM2S confidence predictions, for each sample, across all MB subtypes (WNT, SHH, Group, Group4, as well as Normal samples). Darker colors indicate a higher confidence and greater probability that a given sample belongs to a respective subtype. The *PCARender* function presents PCA plots of tested samples (purple) against the human training set (colored by subtype). This shows, in 3-dimensional space, the nearest MB samples to a given test sample, which indicates how the finalized subtype was assigned using the KNN algorithm. Subtype-centric views include *PredictionsDistributionPie*, which presents a pie charts of the major subtypes predicted across all the samples tested. *PredictionsDistributionBoxplot* highlights overall strength (in terms of MM2S confidence interval) of subtype predictions that were identified across all samples tested

We have developed two main functions (***MM2S.human*** and ***MM2S.mouse***) that apply the MM2S algorithm towards human primary tumours and cell lines, and MB mouse models, respectively (Fig. 1). We ensured a standardized output format that facilitates graphical rendering of the MM2S predictions in a variety of contexts (Fig. 1). We have introduced multiple functions that combine both sample-centric and subtype-centric views of the MM2S output. The sample-centric views (using the functions ***PredictionsHeatmap***, ***PredictionsBarplot*** and ***PCARender***) are easily interpretable and facilitate association of a particular Human MB subtype to normalized gene expression values for a given sample. High-confidence predictions (≥80 % of votes) are indicative of a corresponding human subtype, and lower predictions indicate an intermediate genotype. Where a large number of sample replicates are tested simultaneously, subtype-centric views (using the functions ***PredictionsDistributionPie*** and ***PredictionsDistributionBoxplot***) indicate the majority subtype and consensus predictions across all replicates.

## Results and discussion

We have selected some examples from our previous analysis [4], to demonstrate the data reproducibility and improved data rendering capabilities of the MM2S package compared to the server implementation. MM2S is applied in two case studies involving human primary patient samples and sample replicates of the GTML mouse model. The package and underlying functions we present here are fully documented, easy to install and to incorporate into larger Medulloblastoma-driven analysis pipelines (Additional file 2: Data 1, Additional file 3: Data 2).

### MM2S Prediction of Human MB Subtypes for Patient Tumour Samples

We tested here MM2S on a dataset of human patient samples from the Gene Expression Omnibus (GEO), for which subtypes are already known. The GSE37418 dataset contains 76 primary patient samples including WNT (*n* = 8), SHH (*n* = 10), Group3 (*n* = 16) and Group4 (*n* = 39), and outlier samples not pertaining to the major MB subgroups (*n* = 3). Using the ***MM2S.human*** function, MM2S accurately predicts patient samples across well-studied MB subtypes (WNT and SHH, 100 % accuracy), as well as the lesser-characterized Group3 (87.5 %) and Group4 (79.4 %) (Additional file 4: Table S1, Additional file 5: Table S2). The full code is provided in package vignette and in Additional file 2: Data 1. We also provide additional examples of how to process the data from NCBI GEO prior to using the *MM2S.human* function in Additional file 3: Data 2.

### MM2S Prediction of Human MB Subtypes for the GTML Mouse Model

Using MM2S, we previously identified two genetically engineered mouse models recapitulating transcriptomic patterns of WNT and Group3 subtypes [4]. We expanded here on MM2S predictions using 20 sample replicates of the GTML mouse model. Using the ***MM2S.mouse*** function, we observed the largest number of Group3 predictions across sample replicates (Additional file 6: Table S3). A heatmap representation of MM2S predictions across GTML replicates indicates that the majority of replicates predict as Group3 with high degrees of confidence (>80 %). This is further affirmed by looking at the distribution of predicted subtypes, and the predicted strengths of all the subtype calls, across all the replicates predicted (Additional file 2: Data 1). Overall, our analysis suggests the potential for a non-SHH mouse model but cautions that some of the sample replicates tested also predict as SHH or “normal-like”. These “normal-like” samples are tumour samples that resemble normal cerebellum more than any of the four MB subtypes. Further investigations will need to be conducted on these heterogeneous samples to assess their validity for use as a Group3 mouse model.

## Conclusion

We have implemented the MM2S software package for personalized classification of individual Medulloblastoma (MB) samples from human patients and corresponding model systems into published human MB subtypes**.** We demonstrate the relevance of MM2S to produce robust human subtype classifications for individual human patient samples, and for single-sample replicates of mouse medulloblastoma models. We highlight how our package facilitates single-sample predictions and further investigation into ambiguous genotype potentially due to tumor heterogeneity. The overall design of the MM2S packages makes it a flexible software tool for use by researchers, which would facilitate and extend the use of the MM2S in diverse computational and bioinformatics contexts.

## Availability and requirements

Project Name: MM2S

Project Home Page: The R package MM2S is open source and available on CRAN <https://cran.r-project.org/web/packages/MM2S/> under the GPL-3 License. (Package source code is also available on Github at https://github.com/DGendoo and https://github.com/bhklab).

Operating System: Platform Independent

Programming Language: R

License: GPL-3

## Additional files

Additional file 1: Figure S1. Overall analysis design of the MM2S algorithm. A detailed explanation of the algorithm is provided in the Implementation section of the manuscript. (PDF 894 kb) Additional file 2: Data 1. Vignette demonstrating installation and use of the MM2S package, as well as the code used to reproduce the case studies presented in the manuscript. (PDF 574 kb) Additional file 3: Data 2. Vignette demonstrating how to obtain raw gene expression profiles (from Microarray data) from the NCBI GEO repository, and subsequently filter and normalize the data so that it is ready to use in the *MM2S.human* or *MM2S.mouse* functions. (PDF 208 kb) Additional file 4: Table S1. MB predictions for human patients of the GSE37418 dataset. The final subgroup prediction of MM2S, per sample, is indicated as ‘MM2S_prediction’. Sample predictions are colored by their predicted MB subgroup as follows: Group3 (yellow), SHH (red), WNT (blue), Group4 (green), and normal (grey). For each sample, the closest 5 human neighbors used to make a prediction are also indicated, with percentages indicating the abundance of each particular MB subgroup within the neighbors. (XLSX 15 kb) Additional file 5: Table S2. Confusion matrix of Human MB subgroup predictions for the GSE37418 dataset. Each column of the matrix represents the number of samples in a predicted Human MB subgroup, while each row represents the number of subgroup-specific human samples. (DOCX 15 kb) Additional file 6: Table S3. MB predictions for replicates of the GMTL mouse model from GSE36594. The final subgroup prediction of MM2S, per sample, is indicated as ‘MM2S_prediction’. Sample predictions are colored by their predicted MB subgroup as follows: Group3 (yellow), SHH (red), WNT (blue), Group4 (green), and normal (grey). For each sample, the closest 5 human neighbors used to make a prediction are also indicated, with percentages indicating the abundance of each particular MB subgroup within the neighbors. (XLSX 12 kb)

## Abbreviations

MB: medulloblastoma
MM2S: medullo-model to subtypes
