# Supplementary material for: MM2S: personalized diagnosis of medulloblastoma patients and model systems
Source: Source Code Biol Med. 2016 Apr 11;11:6. doi: 10.1186/s13029-016-0053-y (PMC4827218; doi:10.1186/s13029-016-0053-y)

MM2S CLASSIFIER DESIGN

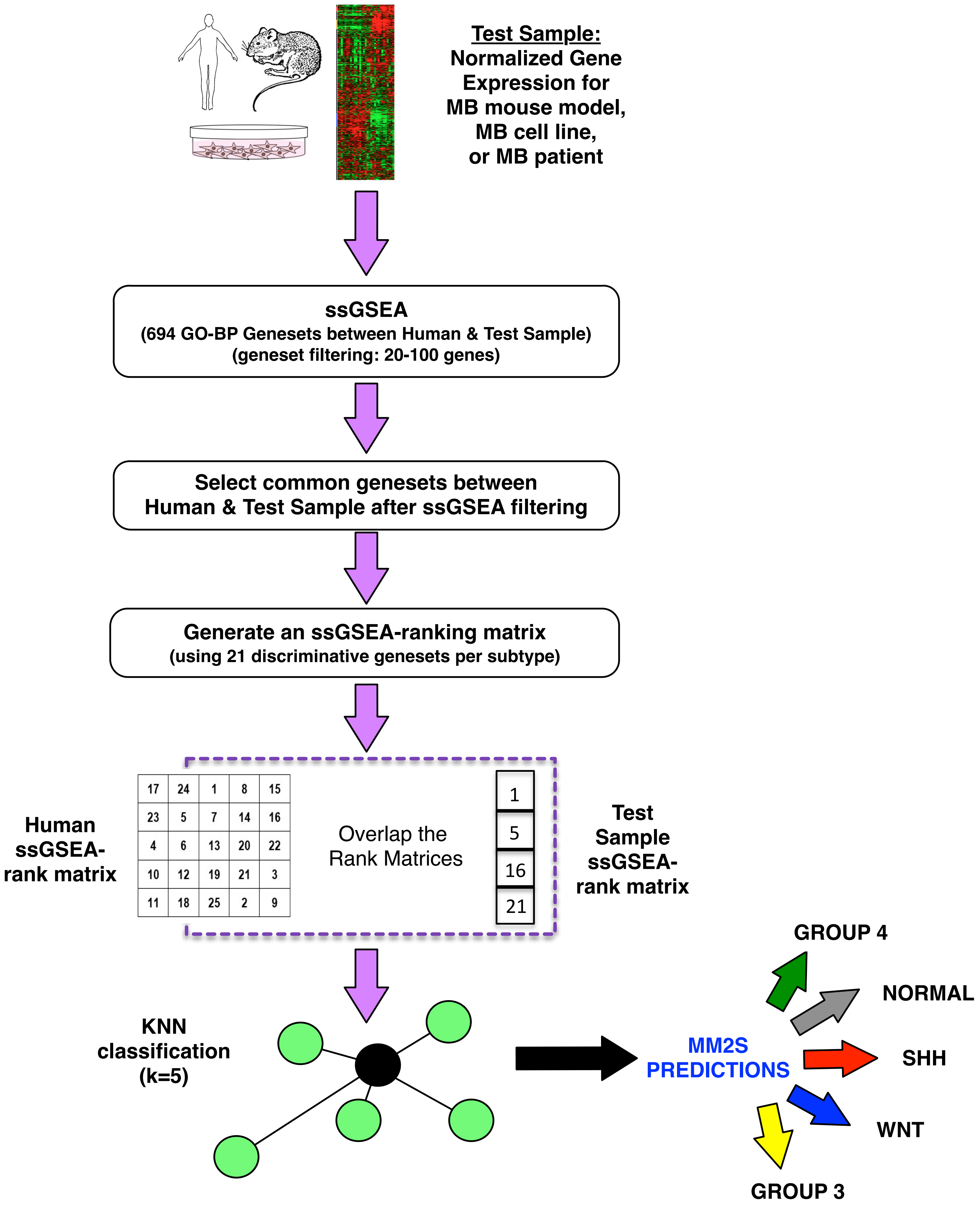

Supplement: Additional file 1: Figure S1. — Overall analysis design of the MM2S algorithm. A detailed explanation of the algorithm is provided in the Implementation section of the manuscript. (PDF 894 kb) [file 13029_2016_53_MOESM1_ESM.pdf]
