# Supplementary material for: MM2S: personalized diagnosis of medulloblastoma patients and model systems
Source: Source Code Biol Med. 2016 Apr 11;11:6. doi: 10.1186/s13029-016-0053-y (PMC4827218; doi:10.1186/s13029-016-0053-y)
Supplement: Additional file 2: Data 1. — Vignette demonstrating installation and use of the MM2S package, as well as the code used to reproduce the case studies presented in the manuscript. (PDF 574 kb) [file 13029_2016_53_MOESM2_ESM.pdf]

MM2S: a package for Medulloblastoma Subtype Predictions

Deena M.A. Gendoo*[1,2] and Benjamin Haibe-Kains†[1,2]

[1]Bioinformatics and Computational Genomics Laboratory, Princess Margaret Cancer Center, University Health Network, Toronto, Ontario, Canada
[2]Medical Biophysics Department, University of Toronto, Toronto, Ontario, Canada

February 24, 2016

Contents

1 Introduction 1

2 Loading package for case studies 2

3 Case Study 1: Predicting Human Subtype Counterparts for Mouse Models 2

4 Case Study 2: Predict Human Subtypes for Primary Patient Samples 10

5 References and Extra Notes 16

6 License 16

7 Session Info 16

1 Introduction

The MM2S package is providing relevant functions for subtype prediction of Medulloblastoma primary samples, mouse models, and cell lines.

MM2S is single-sample classifier that generates Medulloblastoma (MB) subtype predictions for single-samples of human MB patients and model systems, including cell lines and mouse-models. The MM2S algorithm uses a systems-based methodology that faciliates application of the algorithm on samples irrespective of their platform or source of origin. MM2S demonstrates >96 percent accuracy for patients of well-characterized normal cerebellum, WNT, or SHH subtypes, and the less-characterized Group4 (86 percent) and Group3 (78.2 percent). MM2S also enables classification of MB cell lines and mouse models into their human counterparts.This package contains function for implementing the classifier onto human data and mouse data, as well as graphical rendering of the results as PCA plots and heatmaps.

Please refer to the manuscript URL: http://www.sciencedirect.com/science/article/pii/S0888754315000774

Please also refer to the References section for additional information on downloading the MM2S package from Github, or running the MM2S server from the Lab website.

*deena.gendoo@utoronto.ca
†benjamin.haibe.kains@utoronto.ca

2 Loading package for case studies

First we load the MM2S and MM2Sdata packages into the workspace. Both packages are publicly available and can be installed from Bioconductor version 2.8 or higher in R version 2.13.0 or higher.

The MM2Sdata package contains companion datasets that will be used for the examples in the following case studies. The MM2Sdata package contains ExpressionSet objects of both Human and Mouse model Medulloblastoma, specifically:

GSE36594expr: Gene expression for 20 GTML Medulloblastoma mouse samples.

GSE37418Expr: Gene expression for 10 primary Medulloblastoma human samples

Please consult the manual of the MM2Sdata package for more details.

```
#install.packages("MM2S", repos="http://cran.r-project.org")
suppressPackageStartupMessages(library(MM2S))
#install.packages("MM2Sdata", repos="http://cran.r-project.org")
suppressPackageStartupMessages(library(MM2Sdata))
```

3 Case Study 1: Predicting Human Subtype Counterparts for Mouse Models

We first load the Mouse model dataset from GSE36594. We select all samples pertaining to the GTML mouse model. There are 20 sample replicates for this mouse model, all of which are labelled as GTML in the GEO series. We select for those samples and perform MM2S predictions on them.

```
data(GSE36594Expr)
ExprMat<-exprs(GSE36594Expr)
GTML<-ExprMat[,grep("GTML_MB",(colnames(exprs(GSE36594Expr))))]

#Change mouse sample names for clarity
for(sample in 1:ncol(GTML))
{
  newnames<-strsplit(x=(colnames(GTML)[sample]),split="_")[[1]][1]
  colnames(GTML)[sample]<-newnames
}

# Conduct Subtype Predictions for those particular replicates, save results in a XLS file
GTMLPreds<-MM2S.mouse(InputMatrix=GTML,xls_output=TRUE,parallelize=1)

## There are 634 common genesets between Human MB and the Test Data.
## Of these, 106 feature-selected genesets are being used for classification
##
##
## OUTPUT OF MM2S:

##       SampleName MM2S_Prediction Gr3_Confidence Gr4_Confidence
##   [1,] GSM897252  Group3          80             0
##   [2,] GSM897253  SHH             20             0
##   [3,] GSM897254  Group3          80             20
##   [4,] GSM897255  Group3          80             20
##   [5,] GSM897256  SHH             40             0
##   [6,] GSM897257  Group3          60             40
```

```
##  [7,] GSM897258  Group3          80            0
##  [8,] GSM897259  SHH             20            0
##  [9,] GSM897260  SHH             20            0
## [10,] GSM897261  Group3          60            0
## [11,] GSM897262  SHH             20            0
## [12,] GSM897263  SHH             20            0
## [13,] GSM897264  Group3          80            0
## [14,] GSM897265  Group3          60            0
## [15,] GSM897266  Group3          80            0
## [16,] GSM897267  Group3          80            0
## [17,] GSM897268  Group3          60            0
## [18,] GSM897269  Group3          80            0
## [19,] GSM897270  SHH             40            0
## [20,] GSM897271  NORMAL          0             20
##       Normal_Confidence SHH_Confidence WNT_Confidence Neighbor1 Neighbor2
##  [1,] 0                 20             0              SHH       Group3
##  [2,] 0                 80             0              SHH       SHH
##  [3,] 0                 0              0              Group3    Group4
##  [4,] 0                 0              0              Group3    Group3
##  [5,] 0                 60             0              Group3    SHH
##  [6,] 0                 0              0              Group3    Group4
##  [7,] 0                 20             0              Group3    SHH
##  [8,] 0                 80             0              SHH       SHH
##  [9,] 0                 80             0              SHH       SHH
## [10,] 0                 40             0              Group3    SHH
## [11,] 0                 80             0              SHH       SHH
## [12,] 0                 80             0              SHH       Group3
## [13,] 0                 20             0              Group3    Group3
## [14,] 0                 40             0              Group3    Group3
## [15,] 0                 20             0              Group3    Group3
## [16,] 0                 20             0              Group3    SHH
## [17,] 0                 40             0              Group3    SHH
## [18,] 0                 20             0              SHH       Group3
## [19,] 0                 60             0              SHH       SHH
## [20,] 80                0              0              NORMAL    Group4
##       Neighbor3 Neighbor4 Neighbor5
##  [1,] Group3    Group3    Group3
##  [2,] SHH       SHH       Group3
##  [3,] Group3    Group3    Group3
##  [4,] Group3    Group3    Group4
##  [5,] SHH       Group3    SHH
##  [6,] Group3    Group3    Group4
##  [7,] Group3    Group3    Group3
##  [8,] Group3    SHH       SHH
##  [9,] SHH       SHH       Group3
## [10,] Group3    SHH       Group3
## [11,] SHH       Group3    SHH
## [12,] SHH       SHH       SHH
## [13,] SHH       Group3    Group3
## [14,] SHH       SHH       Group3
## [15,] SHH       Group3    Group3
## [16,] Group3    Group3    Group3
## [17,] Group3    Group3    SHH
```

```
## [18,] Group3    Group3    Group3
## [19,] SHH       Group3    Group3
## [20,] NORMAL    NORMAL    NORMAL
```

Now we can view the predictions for the GTML sample replicates in more detail. We first generate heatmap of MM2S confidence predictions for each sample replicate.

```
# Now generate a heatmap of the predictions and save the results in a PDF file.
# This indicates MM2S confidence perdictions for each sample replicate of the GMTL model.
# We view the samples here.
PredictionsHeatmap(InputMatrix=GTMLPreds$Predictions[1:20,],pdf_output=TRUE,pdfheight=12,pdfwidth=10)
```

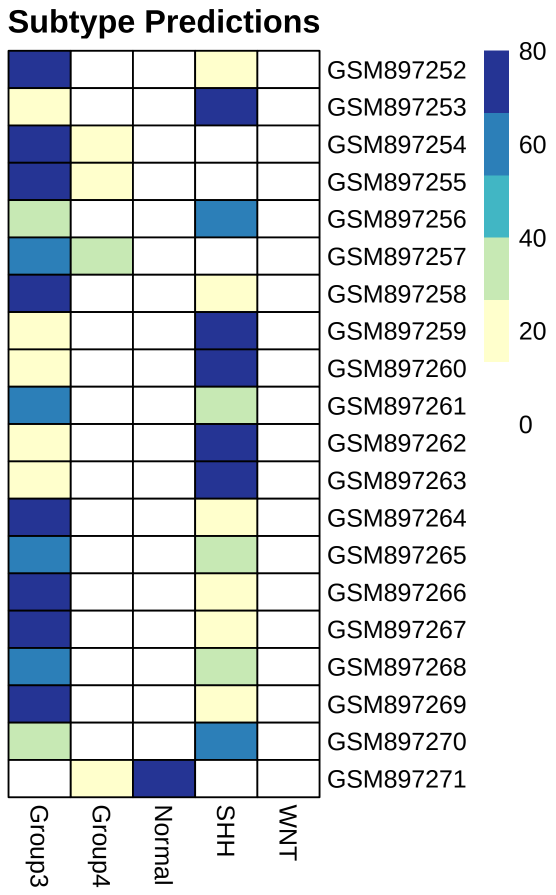

```
# NB: Output may appear on multiple pages
```

We can also represent the results as a stacked barplot.

```
# To run the function all the GTML sample replicates, please run:
# PredictionsBarplot(InputMatrix=GTMLPreds$Predictions[1:20,],pdf_output=TRUE,pdfheight=5,pdfwidth=12)
# NB: Output may appear on multiple pages
```

Using the heatmap or the stacked barplot, we observe that the majority of sample replicates strongly predict as Group3, suggesting the potential for a non-SHH mouse model.

We generate here an overview of the majority subtypes, across all sample replicates. To do this we generate a pie chart of all the predictions.

```
PredictionsDistributionPie(InputMatrix=GTMLPreds,pdf_output=TRUE,pdfheight=5,pdfwidth=5)
```

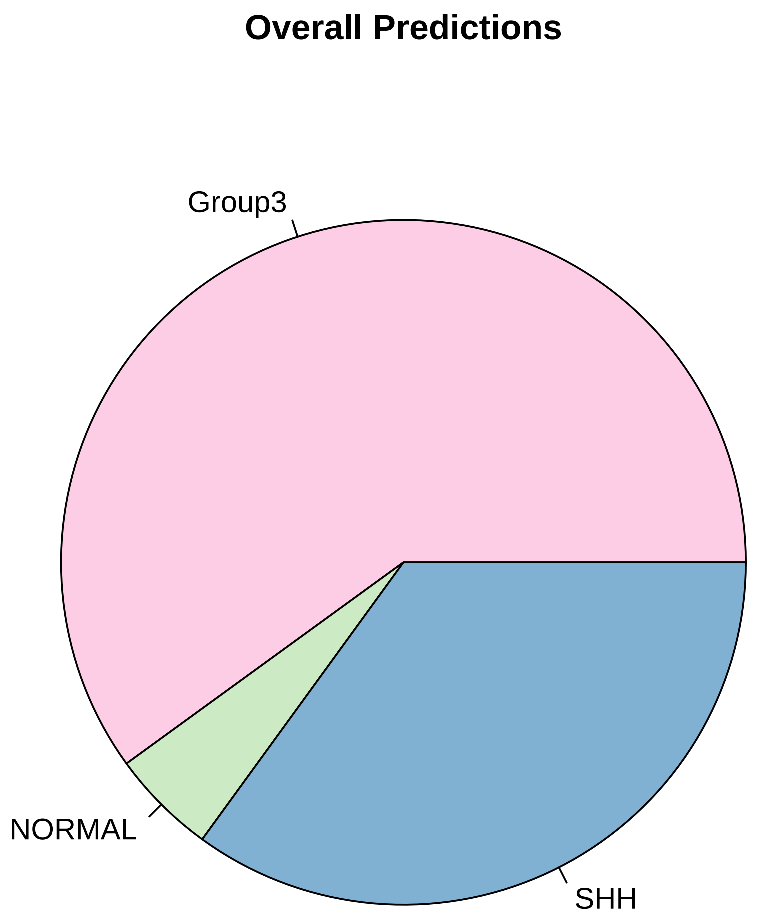

To assess further, we also plot the distribution of subtype calls, across all the samples.

```
PredictionsDistributionBoxplot(InputMatrix=GTMLPreds,pdf_output=FALSE)
```

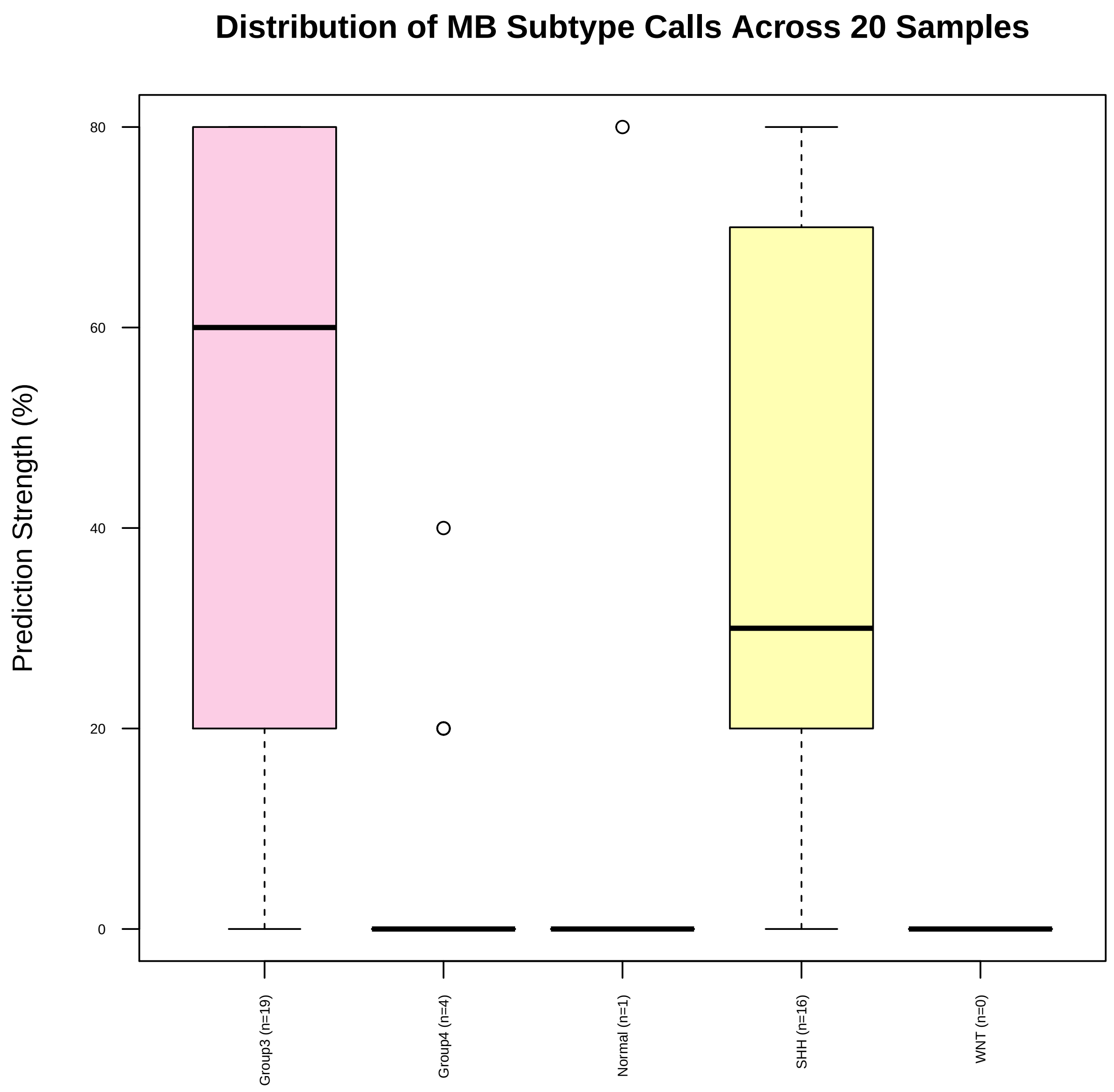

Notably, some samples also predict as either SHH or Normal. Further investigation would need to performed on these samples. To invesitage further, we can graphically visualize different sample replicates and their nearest human MB neighbors from the MM2S training set using Principal Component Analysis (PCA).

Three PDF files are generated which renders PC1 vs PC2, PC2 vs PC3, and a lattice plot of PC1-PC3.

```
PCARender(GSVAmatrixTesting=GTMLPreds$RankMatrixTesting,
          GSVAmatrixTraining=GTMLPreds$RankMatrixTraining)

## Three PDFs have been generated, please consult your working directory to find them.
```

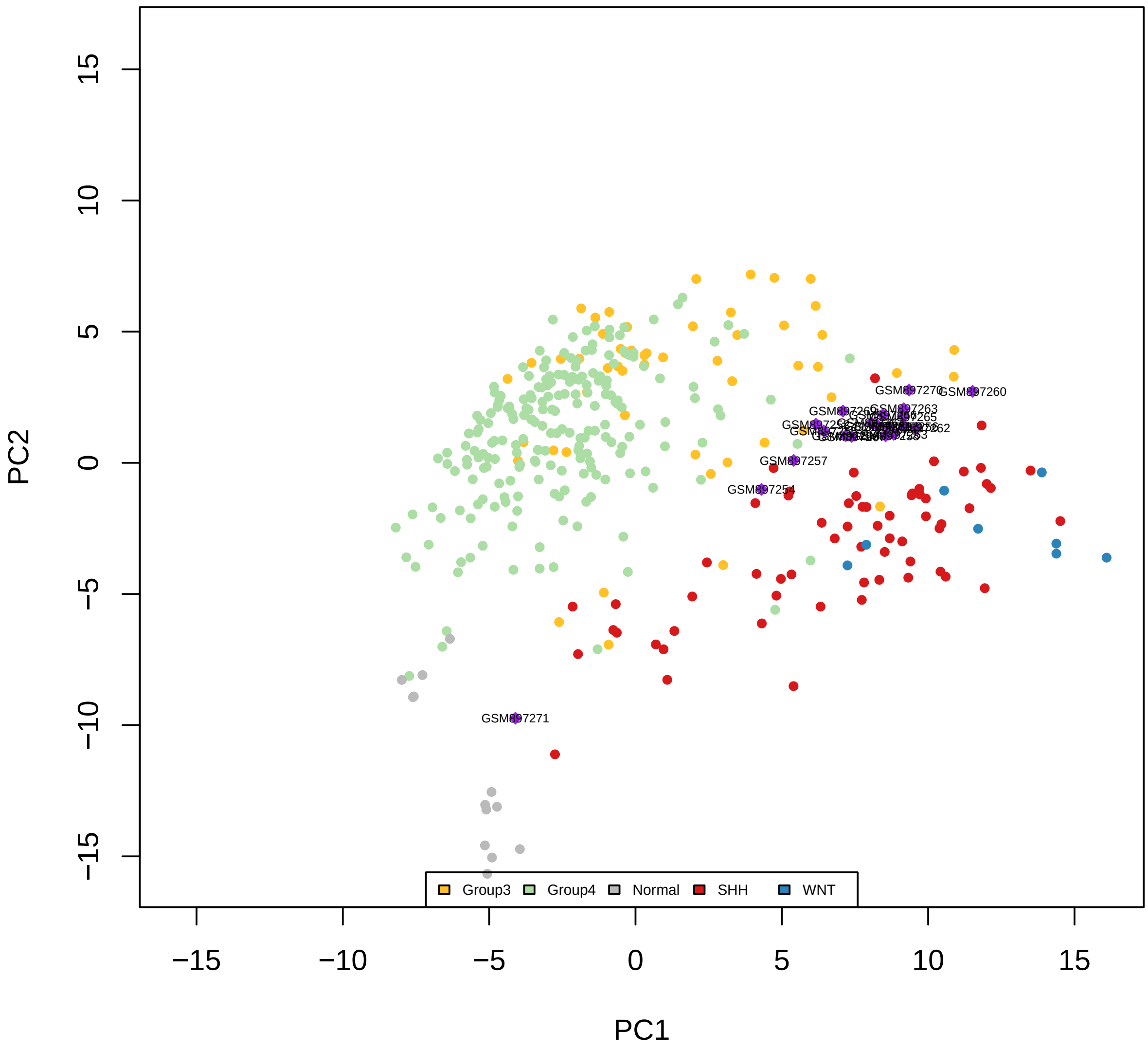
PC2
PC1
15
10
5
0
−5
−10
−15
−15
−10
−5
0
5
10
15
Group3
Group4
Normal
SHH
WNT
GSM897270
GSM897260
GSM897263
GSM897257
GSM897254
GSM897271

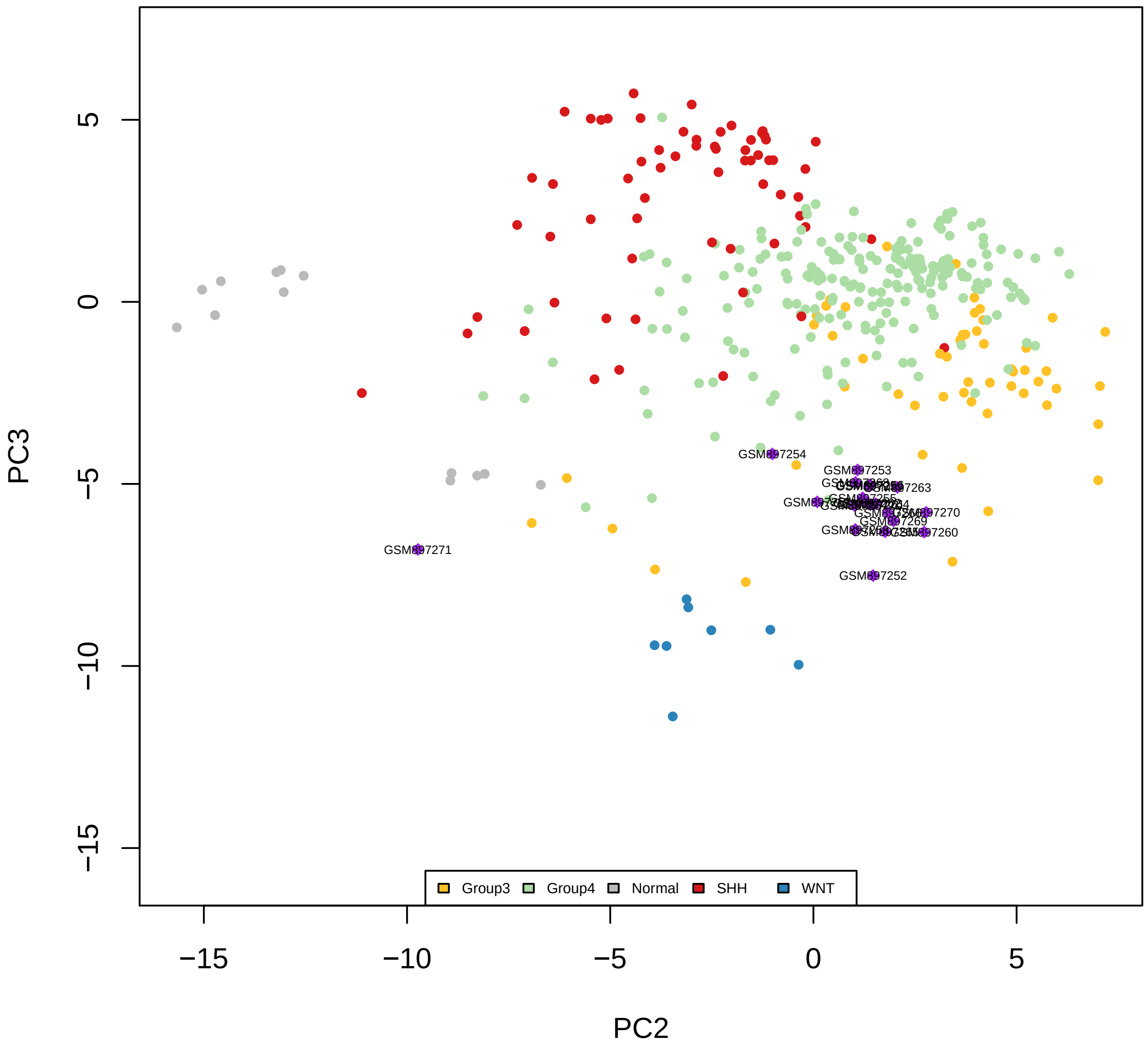
PC3
5
0
−5
−10
−15
PC2
−15
−10
−5
0
5
Group3
Group4
Normal
SHH
WNT
GSM897254
GSM897253
GSM897263
GSM897270
GSM897260
GSM897252
GSM897271

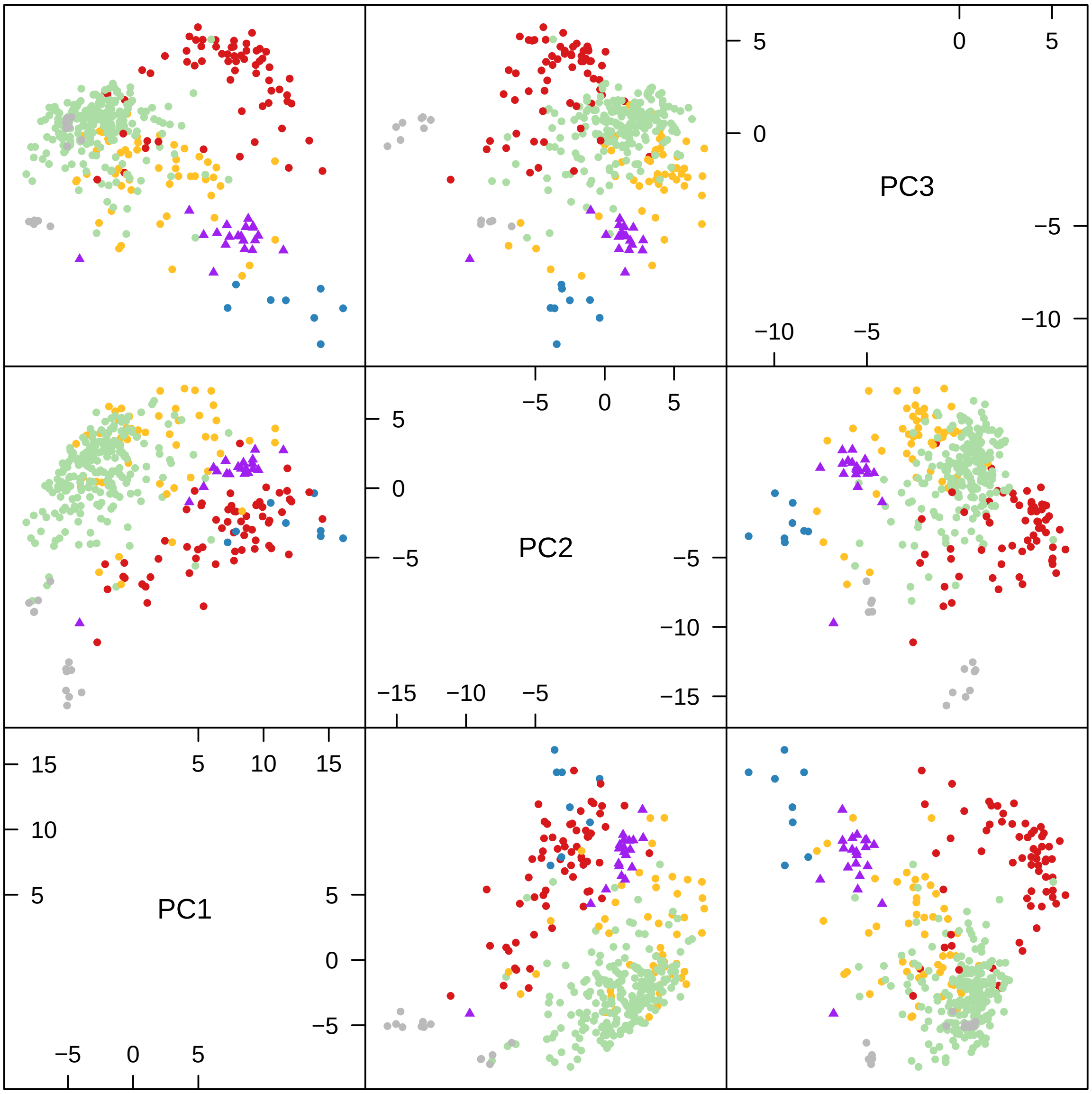

Scatter Plot Matrix

4 Case Study 2: Predict Human Subtypes for Primary Patient Samples

We first load the gene expression data of 10 primary human patient tumours from GSE37418, and conduct MM2S subtype predictions on them.

```
data(GSE37418Expr)
HumanExpr<-exprs(GSE37418Expr)
# Conduct Subtype Predictions for all samples, save results in a XLS file
# [This will take a few minutes to compute]
HumanPreds<-MM2S.human(InputMatrix=HumanExpr,xls_output=TRUE,parallelize=1)

## There are 660 common genesets between Human MB and the Test Data.
## Of these, 105 feature-selected genesets are being used for classification
##
##
## OUTPUT OF MM2S:

##       SampleName               MM2S_Prediction Gr3_Confidence
##  [1,] GSM918580_mbt006-u133v2 WNT             0
##  [2,] GSM918593_mbt035-u133v2 WNT             0
##  [3,] GSM918582_mbt009-u133v2 SHH             0
##  [4,] GSM918606_mbt075-u133v2 SHH             0
##  [5,] GSM918611_mbt085-u133v2 Group4          40
##  [6,] GSM918589_mbt031-u133v2 Group3          100
##  [7,] GSM918624_mbt124-u133v2 Group3          80
##  [8,] GSM918590_mbt032-u133v2 Group4          0
##  [9,] GSM918596_mbt046-u133v2 Group3          60
## [10,] GSM918598_mbt050-u133v2 Group4          20
##       Gr4_Confidence Normal_Confidence SHH_Confidence WNT_Confidence
##  [1,] 0              0                 0              100
##  [2,] 0              0                 0              100
##  [3,] 0              0                 100            0
##  [4,] 0              0                 100            0
##  [5,] 60             0                 0              0
##  [6,] 0              0                 0              0
##  [7,] 20             0                 0              0
##  [8,] 100            0                 0              0
##  [9,] 40             0                 0              0
## [10,] 80             0                 0              0
##       Neighbor1 Neighbor2 Neighbor3 Neighbor4 Neighbor5
##  [1,] WNT       WNT       WNT       WNT       WNT
##  [2,] WNT       WNT       WNT       WNT       WNT
##  [3,] SHH       SHH       SHH       SHH       SHH
##  [4,] SHH       SHH       SHH       SHH       SHH
##  [5,] Group4    Group3    Group4    Group3    Group4
##  [6,] Group3    Group3    Group3    Group3    Group3
##  [7,] Group3    Group3    Group4    Group3    Group3
##  [8,] Group4    Group4    Group4    Group4    Group4
##  [9,] Group4    Group4    Group3    Group3    Group3
## [10,] Group4    Group4    Group4    Group4    Group3
```

We can compare MM2S predictions against known subtype predictions of the samples. These subtype predictions are obtained from the Gene Expression Omnibus (GEO).

```
# We first assess the distribution of the known subtypes for the 76 samples.
table(pData(GSE37418Expr)$characteristics_ch1)

##
##           subgroup: G3           subgroup: G4          subgroup: SHH
##                      3                      3                      2
## subgroup: SHH OUTLIER            subgroup: U          subgroup: WNT
##                      0                      0                      2

# We now assess the distribtuion of MM2S predicted subtypes for the 76 samples.
table(HumanPreds$MM2S_Subtype[,2])

##
## Group3 Group4    SHH    WNT
##      3      3      2      2

# Side-by-side comparison of MM2S predictions and pre-determined subtypes across all samples
# first check that all samples are matching in the pData and MM2S
all(HumanPreds$MM2S_Subtype[,1] == rownames(pData(GSE37418Expr)))

## [1] TRUE

# then generate comparisons
ComparisonTable<-cbind(Sample=rownames(pData(GSE37418Expr)),
                       Original=as.character(pData(GSE37418Expr)$characteristics_ch1),MM2S=HumanPreds$MM
# We view the first 15 samples here
ComparisonTable[1:10,]

##       Sample                      Original        MM2S
##  [1,] "GSM918580_mbt006-u133v2" "subgroup: WNT" "WNT"
##  [2,] "GSM918593_mbt035-u133v2" "subgroup: WNT" "WNT"
##  [3,] "GSM918582_mbt009-u133v2" "subgroup: SHH" "SHH"
##  [4,] "GSM918606_mbt075-u133v2" "subgroup: SHH" "SHH"
##  [5,] "GSM918611_mbt085-u133v2" "subgroup: G3"  "Group4"
##  [6,] "GSM918589_mbt031-u133v2" "subgroup: G3"  "Group3"
##  [7,] "GSM918624_mbt124-u133v2" "subgroup: G3"  "Group3"
##  [8,] "GSM918590_mbt032-u133v2" "subgroup: G4"  "Group4"
##  [9,] "GSM918596_mbt046-u133v2" "subgroup: G4"  "Group3"
## [10,] "GSM918598_mbt050-u133v2" "subgroup: G4"  "Group4"
```

We can easily generate a heatmap of all predictions, as well as PCA plots for our given samples against the MM2S training set.

```
# Now generate a heatmap of the predictions and save the results in a PDF file.
# This indicates MM2S confidence perdictions for each sample.
# We can view the first 10 samples.
PredictionsHeatmap(InputMatrix=HumanPreds$Predictions[1:10,],pdf_output=TRUE,pdfheight=10,pdfwidth=5)
```

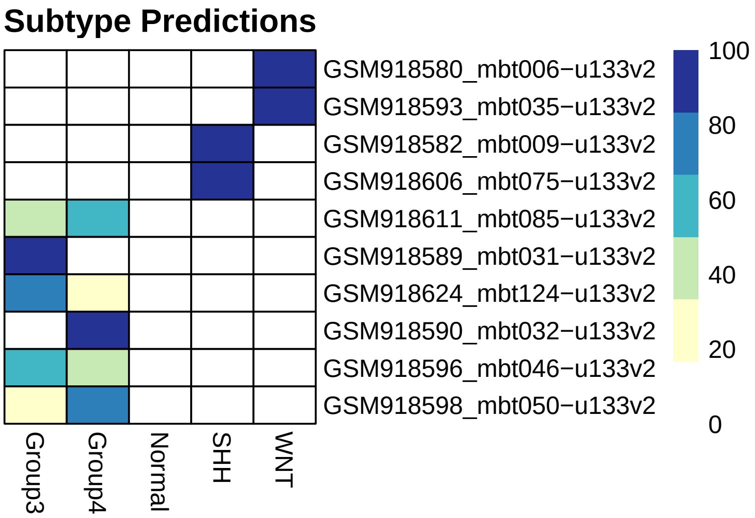


```
# NB: Output may appear on multiple pages

# We can graphically visualize different sample replicates and their nearest human MB neighbors
# from the MM2S training set using Principal Component Analysis (PCA).
PCARender(GSVAmatrixTesting=HumanPreds$RankMatrixTesting,
          GSVAmatrixTraining=HumanPreds$RankMatrixTraining)

## Three PDFs have been generated, please consult your working directory to find them.
```

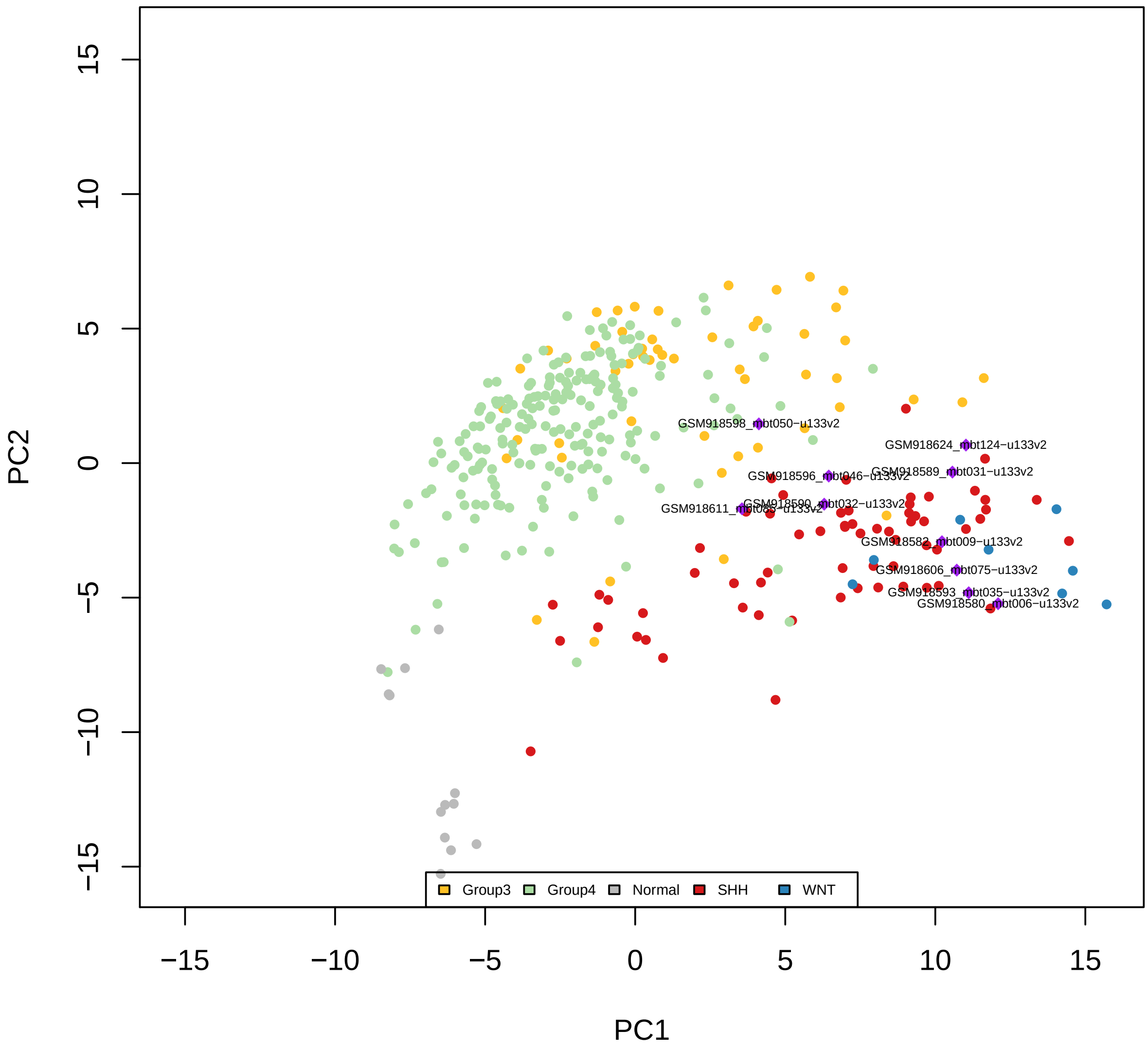
PC2
15
10
5
0
−5
−10
−15
PC1
−15
−10
−5
0
5
10
15
GSM918598_mbt050−u133v2
GSM918624_mbt124−u133v2
GSM918596_mbt046−u133v2
GSM918589_mbt031−u133v2
GSM918590_mbt032−u133v2
GSM918611_mbt085−u133v2
GSM918582_mbt009−u133v2
GSM918606_mbt075−u133v2
GSM918593_mbt035−u133v2
GSM918580_mbt006−u133v2
Group3
Group4
Normal
SHH
WNT

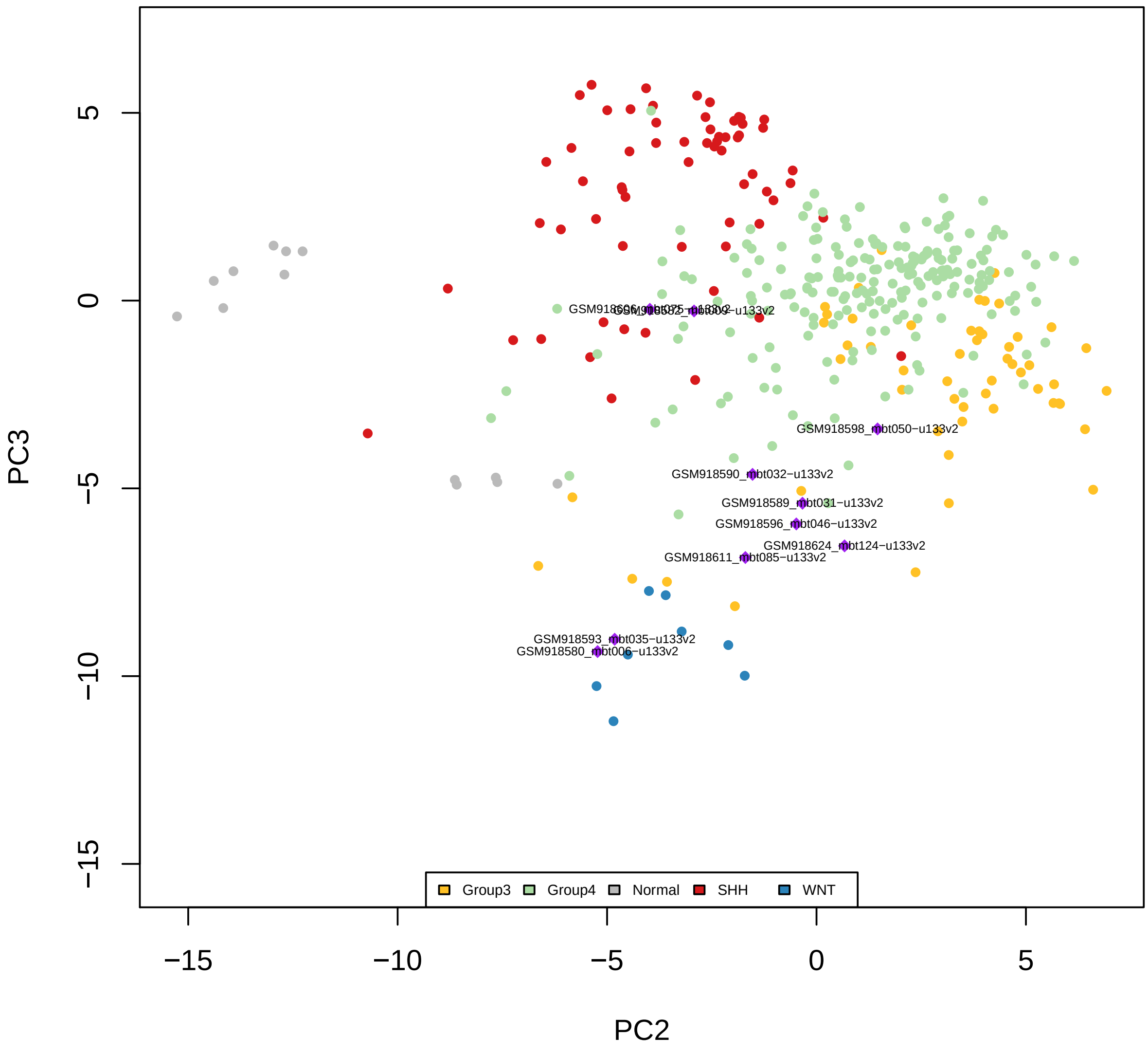
PC3
PC2
5
0
−5
−10
−15
−15
−10
−5
0
5
GSM918598_mbt050−u133v2
GSM918590_mbt032−u133v2
GSM918589_mbt031−u133v2
GSM918596_mbt046−u133v2
GSM918624_mbt124−u133v2
GSM918611_mbt085−u133v2
GSM918593_mbt035−u133v2
GSM918580_mbt006−u133v2
Group3
Group4
Normal
SHH
WNT

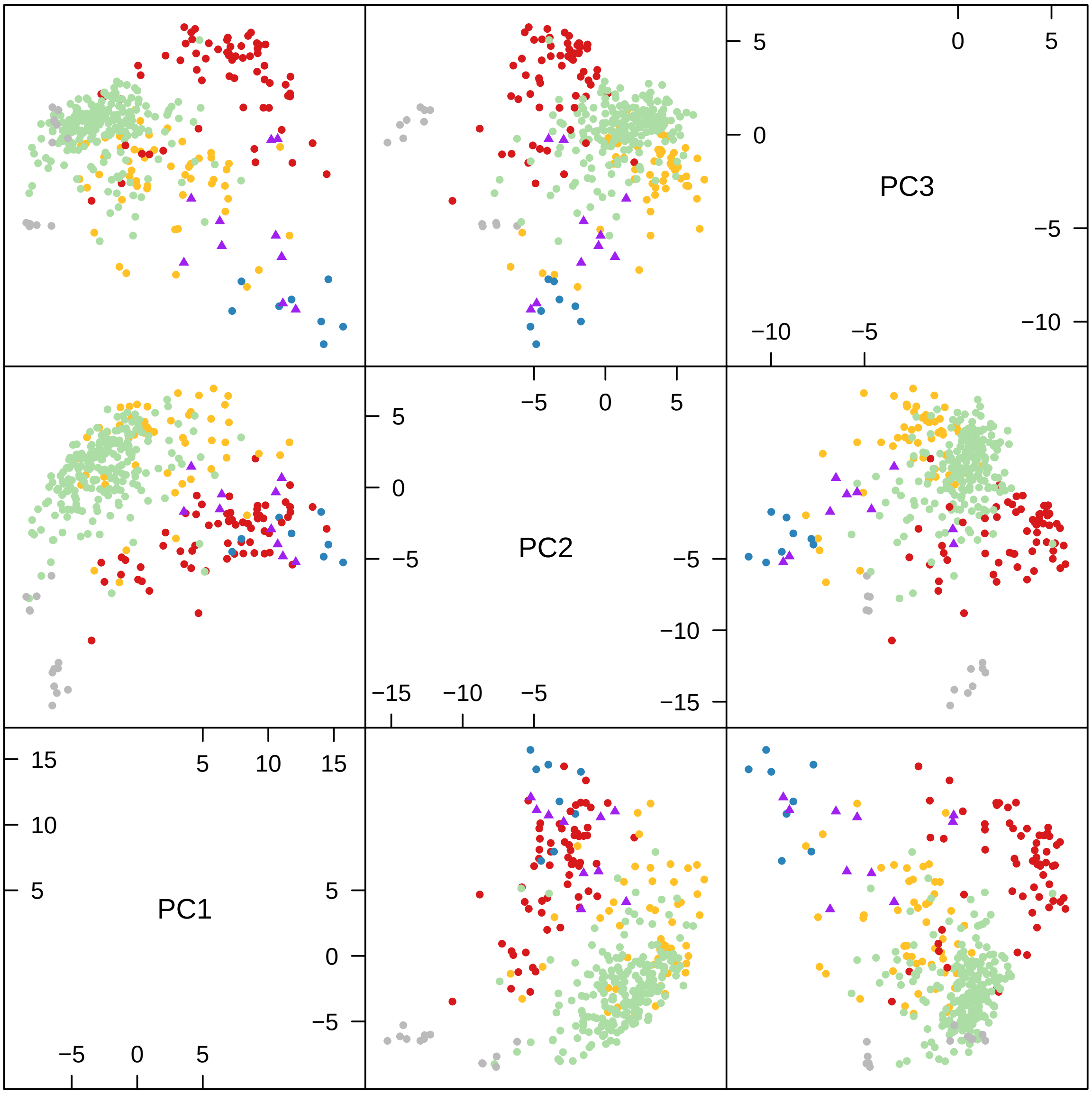

Scatter Plot Matrix

5 References and Extra Notes

Both MM2S and MM2Sdata are publicly available and can be installed in R version 2.13.0 or higher. Both packages are also availabe on Github. Companion datasets are also available on the Haibe-Kains (BHK) Lab website.

Please refer to the following data repositories and websites for additional information, as necessary:

MM2S and MM2Sdata on Github: https://github.com/DGendoo OR https://github.com/bhklab

BHK Lab Website: http://www.pmgenomics.ca/bhklab/software/mm2s

The following code snippet is an example installation of the data repositories from Github.

```
# library(Biobase)
# library(devtools)
# install_github(repo="DGendoo/MM2S")
# install_github(repo="DGendoo/MM2Sdata")
```

6 License

The MM2S package is released under the GPL-3.0 License.

The MM2S package is provided "AS-IS" and without any warranty of any kind. In no event shall the University Health Network (UHN) or the authors be liable for any consequential damage of any kind, or any damages resulting from the use of MM2S.

7 Session Info

- R version 3.2.0 Patched (2015-05-20 r68389), `x86_64-apple-darwin10.8.0`
- Locale: `en_CA.UTF-8/en_CA.UTF-8/en_CA.UTF-8/C/en_CA.UTF-8/en_CA.UTF-8`
- Base packages: base, datasets, graphics, grDevices, methods, parallel, stats, utils
- Other packages: Biobase 2.28.0, BiocGenerics 0.14.0, GSVA 1.16.0, kknn 1.3.0, knitr 1.10.5, lattice 0.20-33, MM2S 1.0.4, MM2Sdata 1.0.1, pheatmap 1.0.7
- Loaded via a namespace (and not attached): annotate 1.46.0, AnnotationDbi 1.30.1, colorspace 1.2-6, DBI 0.3.1, evaluate 0.7, formatR 1.2, GenomeInfoDb 1.4.0, graph 1.46.0, grid 3.2.0, GSEABase 1.30.1, gtable 0.1.2, highr 0.5, igraph 0.7.1, IRanges 2.2.2, magrittr 1.5, Matrix 1.2-0, munsell 0.4.2, plyr 1.8.3, RColorBrewer 1.1-2, Rcpp 0.12.0, RSQLite 1.0.0, S4Vectors 0.6.0, scales 0.2.5, stats4 3.2.0, stringi 0.5-5, stringr 1.0.0, tools 3.2.0, XML 3.98-1.2, xtable 1.7-4
