# Supplementary material for: MM2S: personalized diagnosis of medulloblastoma patients and model systems
Source: Source Code Biol Med. 2016 Apr 11;11:6. doi: 10.1186/s13029-016-0053-y (PMC4827218; doi:10.1186/s13029-016-0053-y)
Supplement: Additional file 3: Data 2. — Vignette demonstrating how to obtain raw gene expression profiles (from Microarray data) from the NCBI GEO repository, and subsequently filter and normalize the data so that it is ready to use in the MM2S.human or MM2S.mouse functions. (PDF 208 kb) [file 13029_2016_53_MOESM3_ESM.pdf]

MM2S: a package for Medulloblastoma Subtype Predictions

Deena M.A. Gendoo*[1,2] and Benjamin Haibe-Kains†[1,2]

[1]Bioinformatics and Computational Genomics Laboratory, Princess Margaret Cancer Center, University Health Network, Toronto, Ontario, Canada
[2]Medical Biophysics Department, University of Toronto, Toronto, Ontario, Canada

February 24, 2016

Contents

1 Introduction **1**

2 Obtaining Microarray Gene Expression Data **1**

3 References and Extra Notes **5**

4 License **5**

5 Session Info **5**

1 Introduction

The MM2S package is providing relevant functions for subtype prediction of Medulloblastoma primary samples, mouse models, and cell lines.

Please refer to the manuscript URL: http://www.sciencedirect.com/science/article/pii/S0888754315000774

Please also refer to the References section for additional information on downloading the MM2S package from Github, or running the MM2S server from the Lab website.

This vignette focuses on downloading and processing a gene expression dataset, and formatting it for use in MM2S.

2 Obtaining Microarray Gene Expression Data

We describe how to obtain and process Gene Expression Microarray Data Human Medulloblastoma Patients from the Gene Expression Omnibus (GEO). These data are downloaded using the GSE Series Number of the Dataset of Interest. For this example, we are downloading GSE37418 dataset, which contains 76 samples of Human MB patients.

First, we need to install several Bioconductor packages and the MM2S package, if they are not already availabe. To properly map probes from gene expression data against their corresponding gene symbols, we rely on BrainArray CDF. This requires an additional download the of CDF package and installation, as well as a modified Affy package. To do so, please run the following commands:

*deena.gendoo@utoronto.ca
†benjamin.haibe.kains@utoronto.ca

```
#Bioconductor package installation
source("http://bioconductor.org/biocLite.R")
biocLite(c("GEOquery","Biobase"))
install.packages("MM2S", repos="http://cran.r-project.org")

#CDF installation
download.file(
  url = "http://mbni.org/customcdf/20.0.0/entrezg.download/hgu133plus2hsentrezgcdf_20.0.0.tar.gz",
              method = "auto",destfile = "hgu133plus2hsentrezgcdf_20.0.0.tar.gz")
install.packages("hgu133plus2hsentrezgcdf_20.0.0.tar.gz",type = "source",repos=NULL)

#Modified Affy package
download.file(
  url = "http://brainarray.mbni.med.umich.edu/Brainarray/Database/CustomCDF/20.0.0/affy_1.48.0.tar.gz",
              method = "auto",destfile = "affy_1.48.0.tar.gz")
install.packages("affy_1.48.0.tar.gz",type = "source",repos=NULL)
```

We now load all the libraries:

```
suppressPackageStartupMessages(library(MM2S))
suppressPackageStartupMessages(library(affy))
suppressPackageStartupMessages(library(Biobase))
suppressPackageStartupMessages(library(GEOquery))
suppressPackageStartupMessages(library(hgu133plus2hsentrezgcdf))
```

Next we download the cel files containing raw expression data into a folder.

```
gse<-getGEOSuppFiles(GEO = "GSE37418")

## ftp://ftp.ncbi.nlm.nih.gov/geo/series/GSE37nnn/GSE37418/suppl/

untar(tarfile = "./GSE37418/GSE37418_RAW.tar",exdir = "CelFiles")
```

We process the downloaded cell files and normalize the gene expression values using BrainArray CDF.

```
# Generate the Affy Expression Object
affyRaw <- ReadAffy(celfile.path = "CelFiles",verbose = F,
                    cdfname="hgu133plus2hsentrezgcdf",compress = T)

# View object
affyRaw

## AffyBatch object
## size of arrays=1164x1164 features (42 kb)
## cdf=hgu133plus2hsentrezgcdf (19425 affyids)
## number of samples=76
## number of genes=19425
## annotation=hgu133plus2hsentrezgcdf
## notes=

#Perform Data Background Correction and Normalization
eset <- expresso(affyRaw,bgcorrect.method="rma",normalize.method="quantiles",
                 pmcorrect.method="pmonly",summary.method="medianpolish",verbose = FALSE)

## 19425 ids to be processed
## |                    |
## |####################|
```

```r
#Obtain the Microarray Expression Dataset
datamatrix<-exprs(eset)

# Polish the rownames (remove the _at from the Entrez IDs)
rownames(datamatrix)<-gsub(rownames(datamatrix),pattern="_at",replacement="")

# Create a new variable representing the cleaned microarray data that will be used in MM2S
ExprMatrix<-datamatrix
```

Now we can use this data and run several MM2S functions to determine the MB subtypes of given samples. We first perform MM2S predictions on a subset of the samples for demonstration.

```r
# Conduct Subtype Predictions the samples, save results in a XLS file
HumanPreds<-MM2S.human(InputMatrix=ExprMatrix[,1:10],xls_output=FALSE,parallelize=4)

## There are 658 common genesets between Human MB and the Test Data.
## Of these, 105 feature-selected genesets are being used for classification
##
##
## OUTPUT OF MM2S:

##       SampleName                     MM2S_Prediction Gr3_Confidence
##  [1,] GSM918578_mbt003-u133v2.CEL.gz Group4          0
##  [2,] GSM918579_mbt004-u133v2.CEL.gz Group4          40
##  [3,] GSM918580_mbt006-u133v2.CEL.gz WNT             0
##  [4,] GSM918581_mbt008-u133v2.CEL.gz Group4          0
##  [5,] GSM918582_mbt009-u133v2.CEL.gz SHH             0
##  [6,] GSM918583_mbt010-u133v2.CEL.gz Group4          20
##  [7,] GSM918584_mbt011-u133v2.CEL.gz Group4          20
##  [8,] GSM918585_mbt013-u133v2.CEL.gz Group4          0
##  [9,] GSM918586_mbt016-u133v2.CEL.gz Group3          100
## [10,] GSM918587_mbt020-u133v2.CEL.gz WNT             20
##       Gr4_Confidence Normal_Confidence SHH_Confidence WNT_Confidence
##  [1,] 100            0                 0              0
##  [2,] 60             0                 0              0
##  [3,] 0              0                 0              100
##  [4,] 100            0                 0              0
##  [5,] 0              0                 100            0
##  [6,] 80             0                 0              0
##  [7,] 80             0                 0              0
##  [8,] 80             0                 20             0
##  [9,] 0              0                 0              0
## [10,] 20             0                 0              60
##       Neighbor1 Neighbor2 Neighbor3 Neighbor4 Neighbor5
##  [1,] Group4    Group4    Group4    Group4    Group4
##  [2,] Group4    Group4    Group3    Group3    Group4
##  [3,] WNT       WNT       WNT       WNT       WNT
##  [4,] Group4    Group4    Group4    Group4    Group4
##  [5,] SHH       SHH       SHH       SHH       SHH
##  [6,] Group3    Group4    Group4    Group4    Group4
##  [7,] Group4    Group4    Group3    Group4    Group4
##  [8,] Group4    Group4    Group4    SHH       Group4
##  [9,] Group3    Group3    Group3    Group3    Group3
## [10,] Group3    WNT       WNT       WNT       Group4
```

Now these predictions can be viewed using a variety of MM2S functions. Here we generate a heatmap of MM2S confidence predictions for the 10 samples we have tested.

```
# Now generate a heatmap of the predictions and save the results in a PDF file.
# This indicates MM2S confidence perdictions for each sample .
# We view the samples here.
PredictionsHeatmap(InputMatrix=HumanPreds$Predictions,pdf_output=TRUE,pdfheight=12,pdfwidth=10)
```

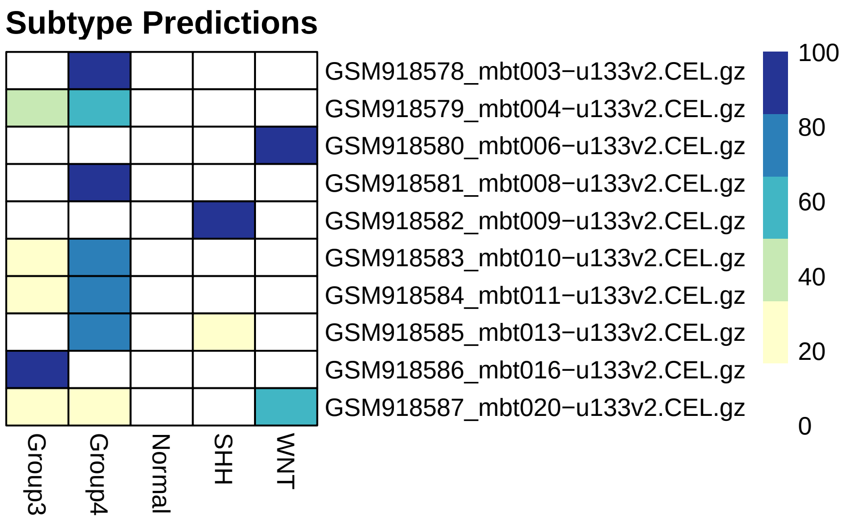

3 References and Extra Notes

Both MM2S and MM2Sdata are publicly available and can be installed in R version 2.13.0 or higher. Both packages are also availabe on Github. Companion datasets are also available on the Haibe-Kains (BHK) Lab website.

Please refer to the following data repositories and websites for additional information, as necessary:

MM2S and MM2Sdata on Github: https://github.com/DGendoo OR https://github.com/bhklab

BHK Lab Website: http://www.pmgenomics.ca/bhklab/software/mm2s

The following code snippet is an example installation of the data repositories from Github.

```
# library(Biobase)
# library(devtools)
# install_github(repo="DGendoo/MM2S")
# install_github(repo="DGendoo/MM2Sdata")
```

A good tutorial on microarray data processing is also available on Biostars: https://www.biostars.org/p/53870/

4 License

The MM2S package is released under the GPL-3.0 License.

The MM2S package is provided "AS-IS" and without any warranty of any kind. In no event shall the University Health Network (UHN) or the authors be liable for any consequential damage of any kind, or any damages resulting from the use of MM2S.

5 Session Info

- R version 3.2.0 Patched (2015-05-20 r68389), `x86_64-apple-darwin10.8.0`
- Locale: `en_CA.UTF-8/en_CA.UTF-8/en_CA.UTF-8/C/en_CA.UTF-8/en_CA.UTF-8`
- Base packages: base, datasets, graphics, grDevices, methods, parallel, stats, stats4, utils
- Other packages: affy 1.48.0, AnnotationDbi 1.30.1, Biobase 2.28.0, BiocGenerics 0.14.0, GenomeInfoDb 1.4.0, GEOquery 2.34.0, GSVA 1.16.0, hgu133plus2hsentrezgcdf 20.0.0, IRanges 2.2.2, kknn 1.3.0, knitr 1.10.5, lattice 0.20-33, MM2S 1.0.4, pheatmap 1.0.7, S4Vectors 0.6.0
- Loaded via a namespace (and not attached): affyio 1.36.0, annotate 1.46.0, BiocInstaller 1.18.5, bitops 1.0-6, colorspace 1.2-6, DBI 0.3.1, evaluate 0.7, formatR 1.2, graph 1.46.0, grid 3.2.0, GSEABase 1.30.1, gtable 0.1.2, highr 0.5, igraph 0.7.1, magrittr 1.5, Matrix 1.2-0, munsell 0.4.2, plyr 1.8.3, preprocessCore 1.30.0, RColorBrewer 1.1-2, Rcpp 0.12.0, RCurl 1.95-4.7, RSQLite 1.0.0, scales 0.2.5, stringi 0.5-5, stringr 1.0.0, tools 3.2.0, XML 3.98-1.2, xtable 1.7-4, zlibbioc 1.14.0
