# Supplementary material for: MM2S: personalized diagnosis of medulloblastoma patients and model systems
Source: Source Code Biol Med. 2016 Apr 11;11:6. doi: 10.1186/s13029-016-0053-y (PMC4827218; doi:10.1186/s13029-016-0053-y)
Supplement: Additional file 5: Table S2. — Confusion matrix of Human MB subgroup predictions for the GSE37418 dataset. Each column of the matrix represents the number of samples in a predicted Human MB subgroup, while each row represents the number of subgroup-specific human samples. (DOCX 15 kb) [file 13029_2016_53_MOESM5_ESM.docx]

**Supplementary Table 2: Confusion matrix of Human MB subgroup predictions for the GSE37418 dataset.**

Each column of the matrix represents the number of samples in a predicted Human MB subgroup, while each row represents the number of subgroup-specific human samples.

| **GSE37418 (n=73*)** | | **Predicted MB Subgroup** | | | | | **Accuracy (%)** |
| --- | --- | --- | --- | --- | --- | --- | --- |
|  |  | **SHH** | **WNT** | **Group3** | **Group4** | **Normal** |  |
| **Actual MB Subgroup** | **SHH (n=10)** | 10 |  |  |  |  | **100** |
|  | **WNT (n=8)** |  | 8 |  |  |  | **100** |
|  | **Group3 (n=16)** |  | 1 | 14 | 1 |  | **87.5** |
|  | **Group4 (n=39)** |  |  | 8 | 31 |  | **79.4** |

* The GSM918618, GSE318628, and GSM918644 have been excluded from this table due to their original designation under GEO as either ‘SHH outlier’ or ‘Undefined’.
